# Changes in Mechanical Properties and Structure of PET Films Treated with Metagenome-Derived LCC^ICCG^ PETase Heterologously Expressed in *Penicillium verruculosum*

**DOI:** 10.3390/polym18121510

**Published:** 2026-06-17

**Authors:** Dmitrii O. Osipov, Alexandra M. Rozhkova, Pavel V. Volkov, Ivan N. Zorov, Olga A. Sinitsyna, Elena S. Trofimchuk, Marina A. Moskvina, Tatyana E. Grokhovskaya, Alexander A. Yaroslavov, Arkady P. Sinitsyn

**Affiliations:** 1 Federal Research Centre «Fundamentals of Biotechnology» of the Russian Academy of Sciences, Moscow 119071, Russia; a.rojkova@fbras.ru (A.M.R.); pvvolkov@mail.ru (P.V.V.); inzorov@mail.ru (I.N.Z.); or apsinitsyn@gmail.com (A.P.S.); 2 Department of Chemistry, M. V. Lomonosov Moscow State University, Moscow 119991, Russia; oasinitsyna@enzyme.chem.msu.ru (O.A.S.); elena_trofimchuk@mail.ru (E.S.T.); moskvina203@yandex.ru (M.A.M.); groch@genebee.msu.ru (T.E.G.); yaroslav@belozersky.msu.ru (A.A.Y.)

**Keywords:** PET, PET recycling, surface erosion, PET crystallinity, LCC^ICCG^ PETase, enzymatic hydrolysis, *Penicillium verruculosum*

## Abstract

This study examines the nature of enzymatic degradation of polyethylene terephthalate (PET) films mediated by a novel recombinant LCC^ICCG^ PETase enzyme preparation based on *P. verruculosum* fungus. The investigation was conducted using amorphous PET samples and PET samples with varying degrees of crystallinity as substrates for PETase-catalyzed hydrolysis under different temperature and pH conditions. Mechanical testing revealed that enzymatic treatment reduced the yield stress by 20–25%, tensile strength by approximately twofold, and elongation at break by 5–10 times, while the deformation mechanism remained unchanged. Enzymatic degradation under acidic conditions was ineffective, whereas increasing the pH to 9–10 markedly accelerated PET degradation and the associated deterioration of mechanical properties. Thermal analysis (TGA, DSC) and microscopy (optical and scanning electron microscopy) demonstrated that degradation was localized at the polymer surface, leading to the formation of cavities, cracks, and submicron-sized pores rather than bulk material disintegration. An inverse correlation was observed between PET crystallinity and susceptibility to enzymatic degradation: samples with crystallinity below 13% could be almost completely degraded, whereas samples with crystallinity above 30% exhibited little or no measurable weight loss over the same period. Low-crystallinity PET underwent rapid degradation accompanied by a transient increase in crystallinity, while highly crystalline PET primarily accumulated surface defects that nevertheless caused a substantial loss of mechanical strength. Consequently, the experimental data obtained in this study provide useful information for understanding PET degradation and for future studies on enzymatic PET recycling. The systematization of feedstock characteristics and the elucidated patterns of enzymatic degradation will enable optimization of pretreatment, enzymatic hydrolysis, and monomer recovery process parameters, thereby facilitating the eventual production of secondary raw materials.

## 1. Introduction

Plastics are constantly and ubiquitously used in our daily lives. With the increasing demand and production rates, the amount of waste generated also grows. However, only a small fraction of global plastic waste is recycled, with recent estimates indicating that approximately 9% of plastic waste is effectively recycled worldwide [1]. Polyethylene terephthalate (PET) is one of the most widely used polymers. It serves as a raw material in the production of packaging and synthetic fibers. PET is a leader in terms of recycling and reuse among other polymers. PET recycling rates vary widely between countries, typically ranging from approximately 15–50%, depending on collection systems, regulatory frameworks, and waste management infrastructure [2,3].

Currently, secondary PET is most often processed using physicochemical methods: mechanical recycling, chemical recycling using acids, alkalis, organic solvents, and pyrolytic decomposition. In addition to requiring harsh conditions such as high temperatures and pressures, aggressive or flammable reagents [4], which entail high capital and energy costs, and the generation of harmful waste, recycling methods do not meet the requirements for raw materials used in food packaging [5]. Most of the recycled PET extracted from bottles is used in lower-quality PET applications. Repeated mechanical recycling inevitably leads to deterioration in the polymer’s consumer properties [6] due to the accumulation of impurities and a decrease in molecular weight.

In this context, the bioprocessing of polymers containing heteroatoms in their main chain, making them prone to hydrolytic degradation, is of particular interest. The most progressive example is the enzymatic recycling of PET which is susceptible to depolymerization by various hydrolytic enzymes, such as esterases. These enzymes produce monomers (terephthalic acid, ethylene glycol, and their esters), which can be reused as raw materials for PET synthesis.

Enzymes that hydrolyze natural and synthetic polymers formed by ester bonds belong to the subclass of esterases within the hydrolase class and differ in their specificity, which is determined by both the structure of the polymers and the enzymes themselves. For instance, chains without bulky side substituents are more likely to be cleaved by enzymes with an active site that has a narrower and less flexible structure, containing more hydrophilic amino acids that interact with the polymer. An example is the cutinase from *Fusarium solani* [1], which degrades cutin and waxes. If the polymer consists of repeating units with bulky and highly hydrophobic substituents, the enzymes acting on it must have a large, flexible, and open active site, with more hydrophobic amino acids. Examples include the recently discovered PETase from *Ideonella sakaiensis* [2] and leaf compost cutinase (LCC) [3]. These enzymes exhibit high activity toward the insoluble polyester PET. The third group includes enzymes that prefer cutin over synthetic polymers as substrates but are still capable of modifying and hydrolyzing PET, especially after appropriate mutations. Examples include various cutinases: ANCUT2 from *Aspergillus niger* [4], TtcutA from *Thielavia terrestris* [5], and TfCut2 from *Thermobifida fusca* [6].

In any case, many PET-degrading enzymes exhibit activity over a wide pH range, but their maximum activity is in a weakly alkaline environment (pH 8–10) and often at elevated temperatures (60–70 °C) [7,8,9], particularly near the glass transition temperature of PET. Conducting hydrolysis at a constant alkaline pH promotes the formation of soluble phthalates rather than poorly soluble terephthalic acid. The use of thermostable enzymes also has several advantages, including reduced risk of contamination of the reaction mixture.

The main industrially significant enzyme preparations used in large-scale production are obtained using microbial producers based on ascomycetes, less often bacterial strains. This is primarily due to the high productivity of the former, i.e., their ability to secrete proteins in quantities sufficient for profitable production. Traditional representatives in this case are fungi of the genera *Trichoderma*, *Penicillium*, *Aspergillus*, *Pichia*, and bacteria such as *Bacillus*, *Clostridia*, and others.

Currently, PETase enzyme preparations are not produced on an industrial scale. On one hand, this is due to the low productivity of the organisms that are the original sources of these enzymes, and on the other hand, to the features that reduce or make it impossible to express heterologous genes from phylogenetically distant organisms.

As a basis for creating enzyme preparations for food and technical purposes, a group of authors developed an expression system based on the filamentous fungus *Penicillium verruculosum* VKM-3972D (currently *Talaromyces verruculosus*) [10]. This system includes a set of vector plasmids, an optimized protoplasting method, as well as transformation and screening methods for recombinant clones. This fungus is a cellulolytic organism, so the main extracellular protein expressed is cellobiohydrolase I (CBHI). The gene of this enzyme is controlled by a strong inducible promoter activated by certain disaccharides in vitro studies which are replaced with cheaper microcrystalline cellulose (MCC) under cultivation conditions. Most PETases reported to date have been produced in bacterial hosts, primarily *E. coli* [11,12], which generally requires intracellular expression followed by cell disruption and protein purification, or yeasts [13,14]. Unlike yeast expression systems, filamentous fungi possess a naturally efficient extracellular protein secretion machinery, enabling the production of large amounts of secreted enzymes directly into the culture medium. In addition, the expression system is based on a strong cellulase promoter induced by inexpensive cellulose-containing substrates. Therefore, achieving a high level of expression of recombinant non-fungal proteins with industrial or medical significance represents an important challenge [15] with significant biotechnological potential. In this context, the present study applies this expression system for the production of a metagenome-derived LCC^ICCG^ PETase fused with CBHI in *Penicillium verruculosum*. This approach enables not only efficient heterologous expression of the enzyme but also its evaluation in the context of PET film degradation, thereby extending the application scope of the fungal expression platform.

## 2. Materials and Methods

### 2.1. Samples of PET Films

#### 2.1.1. Original PET

Initial industrial extruded APET film was obtained from Izolit Trade Ltd. (Moscow, Russia). The APET sample has a thickness of 150 µm, a glass transition temperature of 73 °C, a cold crystallization temperature of 144 °C, and a melting temperature of 247 °C.

#### 2.1.2. Preparation and Pretreatment of PET Films

Semicrystalline samples were obtained by isothermal heating of the initial film in a temperature-controlled cabinet SNOL 58/350 (AB “UMEGA”, Utena, Lithuania) at 110 °C for 15 to 60 min followed by rapid cooling (quenching).

### 2.2. Substrates

p-Nitrophenyl butyrate (pNPB) (Sigma-Aldrich, St. Louis, MO, USA) was a substrate for enzymatic assay.

### 2.3. Plasmid Construction and Recombinant Protein Expression

The overproductive *niaD*-deficient strain *P. verruculosum* F-3972D, obtained from the All-Russian Collection of Microorganisms (VKM, Moscow, Russia), was used as the recipient strain. *Escherichia coli* Mach-1 cells (Invitrogen, Carlsbad, CA, USA) were used for gene cloning and plasmid isolation.

The LCC^ICCG^ PETase gene was amplified using pAL2-LCC plasmid containing a synthetic copy of the codon optimized mutant variant LCC^ICCG^ [16] as a template with the 5′-primer sequence containing part of the linker (flexible or rigid) and the 3′-primer with LIC sequence. The short *cbhi* gene (without linker and cellulose binding module) was amplified using *P. verruculosum* F-3972D gDNA with the 5′-primer with LIC and the 3′-primer containing linker sequence (flexible or rigid). Full chimeric sequence was obtained by overlap PCR using LIC-primers. The PCR product was cloned into pUC-CBHI vector containing *cbhI* promoter and *cbhI* terminator by ligation independent cloning [17].

*P. verruculosum* recombinant strains were obtained by co-transformation via homologous recombination of recipient *P. verruculosum* F-3972D (Δ*niaD*) strain with pCBHI-PET and pSTA10 plasmids according to the CaCl_2_-PEG method [18]. Basic screening was conducted on agar plates with NaNO_3_ and then several positive transformants were grown in 750 mL flasks with 100 mL of media containing MCC (g/L): KH_2_PO_4_ (15), (NH_4_)_2_SO_4_ (5), MgSO_4_∙7H_2_O (0.3), CaCl_2_∙2H_2_O (0.3), yeast extract (10), MCC (40), and wheat bran (10) for 6 days at 32 °C and 220 rpm. Samples of culture broth were tested for esterase activity and protein concentration. The transformant with highest specific activity (U/mg) was selected for growing in 1 L fermenters. Mineral salts and yeast extract were obtained from Dia-M (Moscow, Russia). Microcrystalline cellulose (MCC) was supplied by Accent Microcell Private Ltd. (Ahmedabad, India). Wheat bran was obtained from a local market.

### 2.4. Cultivation of Recombinant Strains and Enzyme Preparations

Recombinant strains were grown in 1 L KF-108/3 fermenters (Prointech LLC, Moscow, Russia) using a nutrient medium of the following composition (g/L), i.e., glucose (40), MCC (40), wheat bran (10), corn extract (30), urea (2.5), KH_2_PO_4_ (14), (NH_4_)_2_SO_4_ (10), CaCl_2_ (0.6), MgSO_4_∙7H_2_O (0.6), at pH 5.0 and 30 °C for 144 h. The *P. verruculosum* strain was fed with glucose and MCC three times during fermentation.

Liquid and fungal biomass were separated using centrifuge (Beckman Coulter, Brea, CA, USA) at 6000 rpm for 30 min, and the obtained supernatant was dried on a Mini Spray Dryer B-290 (Buchi AG, Flawil, Switzerland) to obtain dry enzyme preparation (EP).

### 2.5. Enzyme Activity Assays

For screening purposes positive clones were assayed colorimetrically for esterase activity. The enzyme activity was quantified by determining the amount of p-nitrophenol released by a modification of the procedure described by [19]. The assay mixture contained the 3 mM of pNPB, 100 μL of 0.1 M Na-acetate buffer pH 5.0, and 100 μL of enzyme water solution or cultural liquid. The reaction was performed at 40 °C for 10 min, and stopped by adding 1 mL of 0.5 M phosphate buffer, pH 7.3. One unit of activity is the quantity of enzyme that liberates 1 micromole of *p*-nitrophenol in one minute. Protein concentration was determined according to the Lowry protein assay [20] using BSA as a standard. The protein content in the fractions was measured by OD_280_ on a NanoDrop Lite spectrophotometer (Thermo Fisher Scientific, Waltham, MA, USA).

### 2.6. Protein Purification and Chromatography

Protein of the EP was precipitated with ammonium sulfate (80% of saturation) and then dissolved in 1 mL of 0.05 M Na-acetate buffer, pH 5.0. Subsequent isolation and purification including gel-filtration, anion exchange and hydrophobic chromatography was performed using an ÄKTA pure chromatography system (GE Healthcare Life Sciences, USA) with a UV detector. Gel-filtration was carried out on Bio-Gel P2 column. The desalted protein mixture was then subjected to separation on a Mono Q column (9 ml) in a linear gradient of 1M NaCl (in 20 mM bis-Tris/HCl, pH 6.8) from 0 to 40%. Ammonium sulfate was added to the fractions containing LCC^ICCG^ PETase to a concentration of 1.7 M, and applied to a Source 15Iso column (10 mL). Proteins were eluted in a gradient of 1.7 M ammonium sulfate (50 mM Na-acetate buffer, pH 5.0) from 100 to 0% over 20 column volumes. The mass fraction of the target protein in EP was calculated as the ratio of its mass to the protein mass loaded onto the column.

### 2.7. Enzymatic Hydrolysis of PET Films

Amorphous and semicrystalline PET propeller shaped films of 4 × 1 cm (about 65 mg) were used for pH and temperature optima determination and other weight loss studies as substrate. Experiments were conducted in 15 mL Falcon tubes with 5 mL of desired buffer and LCC^ICCG^ PETase concentration of 0.05 mg mgPET^−1^ at 60 °C and 220 rpm if not mentioned otherwise. The 0.1 M Britton–Robinson buffer pH 3–11 was used to determine the pH-dependence of weight loss, and 0.1 M Tris-HCl pH 8 buffer was used for other studies. The residual weight of PET samples was gravimetrically determined after 24 h, 48 h, 72 h, 96 h, and 120 h with preliminary rinsing of samples with distilled water. Experiments were conducted in triplicate, and the data are presented as mean values.

### 2.8. Quantification of PET Degradation Products

The water-soluble products of PET hydrolysis were determined by high performance liquid chromatography (HPLC). The Agilent 1100 HPLC chromatography system (Agilent Technologies, Santa Clara, CA, USA) includes the following components: a G1376 gradient binary pump, a G1367 auto sampler with a temperature control system, a G1316 column thermostat, a G1315 diode matrix detector, and a Chemstation B.04.04 control and data processing system was used for analysis of PET hydrolysis products. The Kromasil Eternity-5-C18 (4.6 × 250 mm) chromatography column (Nouryon, Surte, Sweden) with the SecurityGuard C18 (4 × 3 mm) precolumn (Phenomenex, Torrance, CA, USA) was selected for this study. The mobile phase consisted of 20% acetonitrile with 10 mM ammonium phosphate, pH 2.5, at a flow rate of 800 µL min^−1^, injection volume 1–3 µL. The detection wavelength was set at 237 nm, and the slit width was configured to 8 nm.

Following PET hydrolysis, the samples were centrifuged at 20,000× *g* for 5 min using an Eppendorf 5425 centrifuge (Eppendorf, Hamburg, Germany). The supernatants were diluted 200–500-fold with the chromatographic mobile phase and transferred to the autosampler for HPLC analysis. Quantitative calibration was performed using external standards of terephthalic acid and ethane-1,2-diol in the concentration range of 50–1000 μg/L. MHET and BHET were identified based on their retention times and UV absorption characteristics by comparison with reference compounds. All measurements were performed in triplicate, and the reported values represent mean results. Quantitative calibration was performed using terephthalic acid (TPA), mono(2-hydroxyethyl) terephthalate (MHET), bis(2-hydroxyethyl) terephthalate (BHET), and ethane-1,2-diol standards in the concentration range of 50–1000 μg/L. Calibration curves exhibited linear responses within the studied range. All samples were analyzed in triplicate.

### 2.9. Differential Scanning Calorimetry (DSC)

Thermophysical properties of polymer samples were studied by DSC method on a Metler TA4000 (DSC-20 cell) (Metler Toledo, Greifensee, Switzerland) in the temperature range of 25–300 °C with a heating rate of 10 °C min^−1^ in a nitrogen atmosphere. The mass of the studied samples was 5–6 mg. The degree of crystallinity (χ_c_, %) of the samples was determined according to the equation:

$$\chi_{c}=\frac{∆H_{m}-∆H_{cc}}{∆H_{m}^{0}}\times 100,$$

where ∆H_m_—experimentally measured enthalpy of melting; ∆H_cc_—experimentally measured enthalpy of cold crystallization; ∆H^0^_m_—theoretical enthalpy of melting of polymer with 100% degree of crystallinity (for PET ∆H^0^_m_ = 140.1 J g^−1^).

### 2.10. Optical and Scanning Electron Microscopy

Structural and morphological studies of PET films were carried out on a Nikon ECLIPSE E400 optical microscope (Nikon, Tokyo, Japan) with a Mintron MTV-63W1P (Mintron Enterprise, New Taipei City, Taiwan) digital camera. The surface and internal structure of film samples were investigated by scanning electron microscopy (SEM) on a JSM-6380LA microscope (JEOL, Akishima, Japan), 20 kV operating voltage. To investigate the volumetric properties the samples were cleaved using the brittle fracture technique in liquid nitrogen. The obtained cuts and surfaces were attached to a special microscopic slide using double-sided conductive carbon tape and coated with a 25 nm platinum layer on an IB-3 Ion Coater (Eiko, Hitachinaka, Japan).

### 2.11. Stress–Strain Test

Mechanical tests were performed on a universal testing machine Z3-X500 (Thümler GmbH, Nürnberg, Germany) with a 50 N Transducer (Nordic Transducer, Hadsund, Denmark) in air at room temperature (23–24 °C). The strain rate was 2.5 mm min^−1^ (25% min^−1^). The test objects were prefabricated double-sided blades made of PET films with a working section 10 mm long and 5.2 mm wide. All sample types were tested in triplicate. Mechanical parameters are expressed as mean ± standard deviation. The thickness of the samples was measured using an MCC-25 digital micrometer, accuracy ±0.001 (Etalon, Shenzhen, China).

## 3. Results and Discussion

### 3.1. Cloning and Expression of LCC Gene in P. verruculosum Host

In previously published works IsPETase and LCC PETase were obtained by expression either in *E. coli* or yeast cells, which did not produce sufficient protein for industrial use of these enzymes. The use of PETase for cost-effective PET recycling is only expected to be possible if it is produced in sufficient quantities and at low cost. Therefore, a promising producing strain of *P. verruculosum* was used in this work, the efficacy of which has been shown previously [21,22,23]. A strain of *P. verruculosum* PET was obtained using an expression system of this fungus based on a strong MCC-inducible promoter of the *cbhI* gene. All our attempts including direct control under the *cbhI* promoter and the addition of introns to the gene, did not result in its expression in *P. verruculosum*. Therefore, chimeric constructs were created. They consist of *cbhI* gene lacking the region encoding the native linker and cellulose-binding domain on C-terminus, and LCC^ICCG^ PETase gene on N-terminus. The two genes were interconnected by two types of synthetic linkers: flexible neutral polyglycine linker (GGGGS)_4_ (FL), previously described in [24,25,26,27], and rigid α-helical linker (EAAAK)_4_ (RL), previously designed by [28,29]. These and similar linkers have previously been used to stabilize, improve folding, and increase the biological activity of various chimeric proteins [30]. Some fusion partners could promote expression as have been demonstrated for *E. coli* [31,32,33], yeasts [34] and mammalian cells [35]. CBHI was employed as a fusion partner to enhance the expression and secretion of the target protein

The expression cassettes containing short *cbhI*, linker and LCC^ICCG^ PETase coding sequence were obtained by overlap PCR. The linker sequence was incorporated into the overlapping ends of the primers. The resulting products approximately 2300 base pairs in size were cloned using LIC into a shuttle vector based on the pUC-19 plasmid, containing the promoter and terminator regions of the *cbh1* gene as flanking elements. These constructs were used for co-transformation of the recipient strain *P. verruculosum* F-3972D (Δ*niaD*) along with the pSTA10 plasmid through homologous recombination, which led to the integration of the target protein gene and the restoration of the fungal strain’s nitrate autotrophy. Selection of transformants was carried out on agar plates with nitrate, followed by cultivation in liquid medium in flasks. During screening it was discovered that some clones successfully expressed and secreted the target protein extracellularly. Unusually, the LCC^ICCG^ PETase appeared as a distinct band on the electrophoretogram rather than in a fusion form with CBHI independently of the linker type. The explanation for this phenomenon likely lies in the fact that acidic extracellular proteases secreted during fungal cultivation in liquid medium degrade the linkers causing the previously linked enzymes to coexist autonomously despite the fact that these linkers were previously considered relatively resistant to proteolytic hydrolysis.

**Figure 1:**
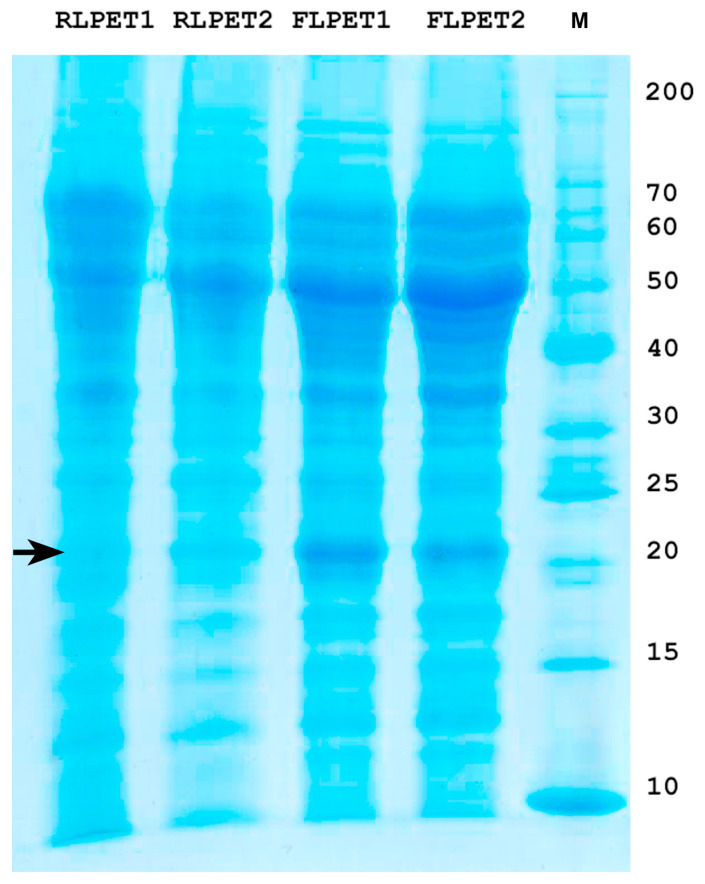
SDS-PAGE of dry recombinant LCC^ICCG^ PETase enzyme preparations. M, molecular weight marker; the expected LCC^ICCG^ PETase band is indicated by an arrow.

Clones selected for the highest protein productivity and pNPB activity were cultivated in 1 L fermenters. The supernatant was separated from the fungal biomass and insoluble medium residues and lyophilized. In total, two transformants were cultivated for each chimera. On the electrophoretogram of the obtained enzyme preparations (EPs) (Figure 1), a distinct band in the range of 23 to 25 kDa is clearly visible. Using chromatography, LCC^ICCG^ PETase was purified (Figure S1) and its content in the EPs was determined. LCC^ICCG^ PETase is eluted from a column with a hydrophobic carrier at an ammonium sulfate concentration of 0.82–0.29 M. The presence of PETase in the fractions was confirmed by SDS-PAGE, mass spectrometry, as well as by high activity toward pNPB. MS analysis showed that peptides with m/z characteristic of LCC^ICCG^ PETase are present not only in the 23 kDa region but also in the 77 kDa region, i.e., where the full-size chimeric protein would be expected. Thus, some amount of the chimeric protein remains intact. Regarding the presence of linkers or their fragments in the free form of LCC^ICCG^ PETase, the MS analysis data suggest the possible presence of a flexible linker residue in the form of GSGGGGSGGGGSGGGGS or GSGGGGSGGGGS. MS analysis of tryptic hydrolysates of bands with the rigid linker did not reveal it in whole or in part, in conjunction with LCC^ICCG^ PETase.

Overall, it turned out that the level of expression using FL is significantly higher than with RL. It was shown that the pNPB esterase activity for FL EPs ranges from 63,900 to 98,700 U/g, while for RL EPs it is 2550–21,200 U/g with the total protein content being 43.5–45.5% and 17–25%, respectively (Table 1).

It should be noted that pNPB hydrolytic activity reflects the catalytic efficiency of the enzymes toward a small, soluble ester substrate and does not directly predict their performance on solid polymeric PET. PET hydrolysis is additionally influenced by interfacial phenomena, including enzyme adsorption and substrate accessibility. Therefore, in the case of using the *P. verruculosum* fungus expression system to produce the target protein, FL demonstrated a higher level of expression, and EPs based on the corresponding recombinant strain were used in the further work. Accordingly, selection of FLPET2 for further experiments was primarily based on expression level and pNPB activity as a rapid screening parameter rather than direct PET degradation performance. The proteolytic instability of the linkers in this case is undoubtedly an advantage, as it allows for the avoidance of the stage of separating the target enzyme from its partner, which could negatively affect the properties of LCC^ICCG^ PETase.

### 3.2. Enzymatic Treatment of PET Films

#### 3.2.1. Effect of Temperature and PETase Concentration on Hydrolysis Rate and Mechanical Properties of APET Films

Initially, the experiments with LCC^ICCG^ PETase (20% of total protein) EP were carried out with 0.75 and 2.25 mg of total protein mg^−1^ PET (0.15 and 0.46 mg LCC^ICCG^ PETase mg^−1^ PET) in 5 mL of 0.1 M Tris-HCl buffer pH 8 at 30 and 60 °C for 24 h. The equivalent concentration of B1 EP solution and amount 0.1 M Tris-HCl buffer pH 8 were used as a control.

After treatment in the buffer and B1 EP solution the appearance of the PET film did not change and remained transparent. Treatment with LCC^ICCG^ PETase solutions resulted in the opacity of the initially amorphous samples. After incubation at 60 °C the film became opaquer (in some cases white). The appearance of opalescence in this case can be associated with both crystallization and formation of caverns (pores) in the process of enzymatic hydrolytic breakdown of the PET ester bond.

**Figure 2:**
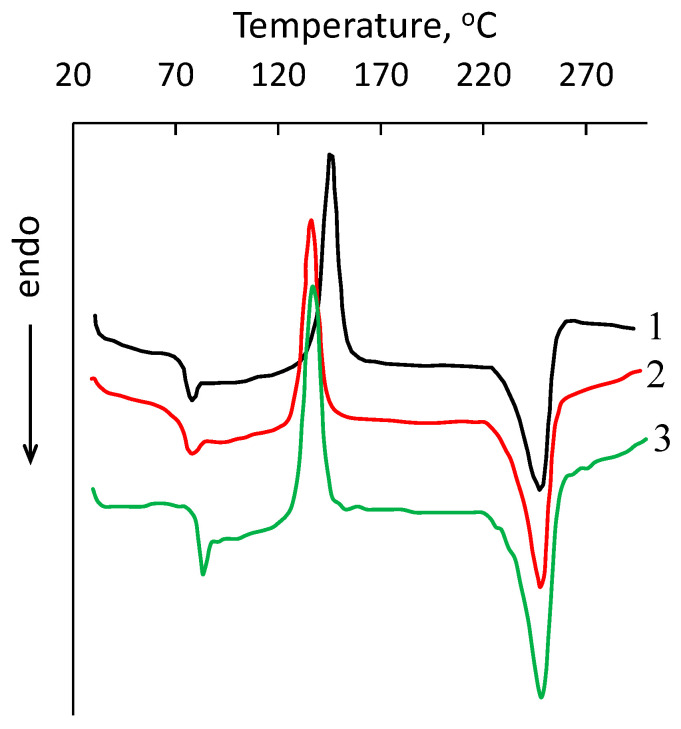
DSC curves of the original APET film (1) and after its incubation in a solution of 0.15 mg LCC^ICCG^ PETase mg^−1^ PET mg for 24 h (2) and 0.03 mg LCC^ICCG^ PETase mg^−1^ PET for 72 h (3) enzyme preparation at 60 °C and pH.

Figure 2 shows the DSC curves of the initial APET film and after its treatment with PETase EP of different concentration for different time. It can be seen that all the curves are similar. They exhibit a step with a small asymmetric endothermic peak in the glass transition temperature region of 73–79 °C, an exothermic peak of low-temperature (“cold”) crystallization at 136–144 °C, and an endothermic melting peak at 247–248 °C. The appearance of the peak in the glass transition temperature region is a consequence of the reduced enthalpy of the polymer sample and is associated with its so-called structural (physical) aging [36]. This aging can lead to improved molecular packing or increased concentration of low-energy conformations over time, even when the polymer is stored at temperatures below the glass transition temperature.

Table 2 shows the peak temperatures and thermal effects of transitions for different PET samples and the degree of crystallinity after treatment with PETase EP solution.

The obtained data indicate that treatment with the LCC^ICCG^ PETase EP, as well as incubation in control solutions (buffer and B1 EP) at 30 °C does not lead to any changes in the glass transition (*T*_g_), cold crystallization (*T*_cc_) and melting temperatures (*T*_m_) of PET. There is only a slight increase in the degree of crystallinity of the polymer up to 4–5% after its treatment with LCC^ICCG^ PETase EP. Conducting the process at 60 °C for 24 h leads to a 10 °C decrease in the peak temperature of cold crystallization. This can be explained by the formation of crystallization nuclei, such as a mesophase, during the polymer’s exposure near the glass transition temperature in model environments. In the presence of LCC^ICCG^ PETase, crystallization nuclei (crystallites) may form as a result of enzymatic degradation, as indicated by an increase in the degree of crystallinity of PET to approximately 9%. Extending the exposure time of PET samples at 60 °C to 72 h results in an increase in the glass transition temperature, which may be a consequence of the physical (structural) aging of the polymer. This is further supported by the increase in the intensity of the asymmetric endothermic peak in the glass transition temperature region.

It is important to note that increasing the enzyme concentration and treatment time does not lead to any significant changes in the thermal properties of the polymer samples, as can be seen from the data in Table 2. This fact can be explained by the gradual surface degradation of the polymer [37]. As a result, changes in the degree of crystallinity are minor and occur only in the near-surface layers. Based on the assumption that the crystalline phase is uniformly distributed throughout the volume of the polymer film during volume crystallization, the volume fraction of the crystalline phase during crystallization from the surface into the depth of the film will be proportional to the thickness of the crystallized layer. If we assume that the densities of amorphous (1.335 g cm^−3^) and crystalline (1.455 g cm^−3^) PET are approximately equal, then *χ*_c_·*L* = *χ*_c_(max)·2*l*_c_, where *χ*_c_ is the experimentally determined degree of crystallinity for the film sample (ranging from 5.5 to 13.5%, based on data in Table 2), *χ*_c_(max) is the most probable maximum degree of crystallinity of PET (about 40% [25]), *L* is the thickness of the starting film (150 μm), and *l*_c_ is the thickness of the crystallized film layer (coefficient 2 takes into account two film surfaces). So, the thickness of the layer where crystallization occurs is approximately 10–25 µm, which corresponds to 7–17% of the total thickness of the original film.

The study of samples using TGA (air atmosphere) showed that the onset temperature of weight loss for the PET film after treatment with LCC^ICCG^ PETase at 60 °C shifts to higher temperatures, from 310 to 323 °C, although the shape of the curve and the peak weight loss temperature remain unchanged. This effect can also be attributed to the crystallization of the surface layers of the film [36], which reduces the rate of oxygen diffusion into the polymer bulk during the initial stage of PET thermo-oxidative degradation.

**Figure 3:**
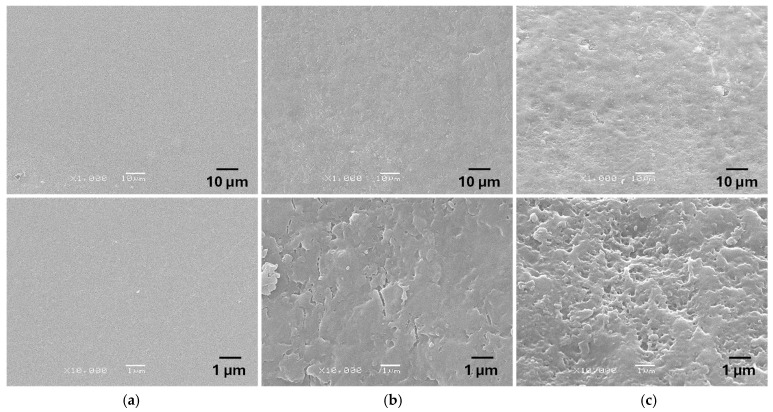
Scanning electron microscope (SEM) micrographs of the APET film surface after incubation with B1 EP (**a**) and LCC^ICCG^ PETase EP solutions with concentrations of 0.03 mg LCC^ICCG^ PETase mg^−1^ PET (**b**) and 0.15 mg LCC^ICCG^ PETase mg^−1^ PET (**c**) at 60 °C for 24 h at 1000× (**top row**) and 10000× (**bottom row**) magnification.

Figure 3 shows SEM micrographs of the surfaces of PET film samples treated with LCC^ICCG^ PETase EP and control (B1 EP) solutions of different concentrations at 60 °C.

After treatment with the control enzyme preparation the sample surface (Figure 3a) remains relatively smooth and free of visible defects. After incubation with LCC^ICCG^ PETase EP the surface of the polymer film becomes rough regardless of LCC^ICCG^ PETase EP concentration. The images (Figure 3b,c) revealed micron-sized cavities (depressions) and cracks, within which submicron-sized pores are observed. The obtained results confirm that the enzyme primarily interacts with the surface of the polymer through a surface erosion mechanism. This process resulted in reductions in both polymer mass and film thickness: the weight loss was approximately 25% and the film thickness decreased by an average of 20% reaching 115–125 µm at pH 8 and 60 °C, after 72 h, regardless of the LCC^ICCG^ PETase EP concentration.

Table 3 presents the mechanical tests data of the initial APET film and after its treatment in B1 EP and LCC^ICCG^ PETase EP enzyme solutions under various conditions. It should be noted that the stress–strain curve (mechanical stress vs. strain) for the initial APET film exhibits a classical shape typical for glassy polymers. At initial strain levels (up to 2–3%), a linear region is observed, where Hooke’s law for elastic bodies is applicable (elastic behavior region). The tangent of the slope of this region corresponds to the elastic modulus, which for the original PET film is approximately 1.2 GPa. Further, the stress–strain curve deviates from linear behavior, and around 5% strain, a yield point appears, characterized by a neck-like sharp local narrowing in the working part of the sample. This is accompanied by a slight decrease in mechanical stress, after which the curve reaches a plateau: a continuous transition of the polymer material into the neck occurs (region of cold drawing) at nearly constant stress up to 340–350% strain. At higher strain levels, when the working part has completely transformed into the neck, the polymer sample again exhibits a linear increase in stress until rupture (orientation hardening region).

Incubation of APET samples in different EP solutions at 30 °C for 24 h had practically no effect on their mechanical properties. For films treated with PETase EP, the elongation at break decreased by an average of 10%. Treatment at 60 °C in air, in 0.1 M Tris-HCl pH 8 buffer, and in the B1 EP solution also did not alter the mechanical properties of PET. However, incubation of the polymer in the presence of LCC^ICCG^ PETase EP for 24 h significantly affected its mechanical properties. A reduction in mechanical stress at the yield point and plateau by 20–25%, a twofold decrease in stress at break (to 30 MPa), and 5–10 times decrease in elongation at break (on average, to 50–130%) were observed. Increasing the treatment time to 72 h, regardless of the LCC^ICCG^ PETase EP concentration, did not lead to a further decrease in the mechanical parameters of PET. Additionally, it should be noted that the deformation mechanism of the polymer film before and after treatment under various conditions remains unchanged and proceeds through the formation and development of a neck. The earlier failure of samples treated with PETase EP solution during the neck development stage may be associated with surface defects caused by the enzymatic degradation of the polymer or as a result of cold crystallization (Figure 3b,c). It is important to note, if the degradation processes affected the bulk of the film (e.g., if pores formed in the volume), this would lead to significant embrittlement and premature fracture at the yield point or during the neck propagation stage.

Thus, the conducted study demonstrated that the degradation process of the APET film at pH 8 proceeds via a surface erosion mechanism. Its rate depends on the process temperature and is practically independent of the PETase EP concentration.

#### 3.2.2. Kinetic Characterization of the PETase Mediated Hydrolytic Degradation of APET Films

**Figure 4:**
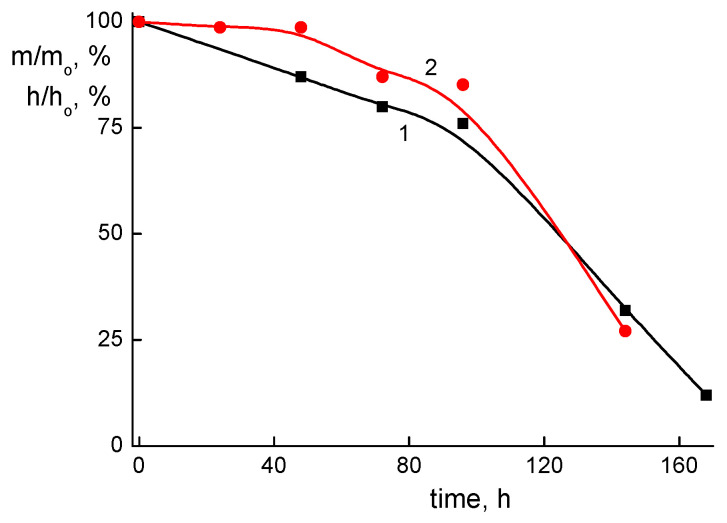
Dependence of residual weight fraction (1) and thickness (2) of APET film on the time of hydrolytic degradation in the solution of PETase EP at 60 °C.

The next experiment was conducted at 60 °C, pH 8, and an initial PETase concentration of 2 mg ml^−1^ (0.07 mg PETase mg^−1^ initial PET). Since enzyme activity in aqueous solution may decrease over time, fresh enzyme was added after 48 h and 96 h, increasing the LCC^ICCG^ PETase concentration to 4 and 6 mg mL^−1^, respectively. Figure 4 shows the time dependencies of the mass and thickness reduction of the APET film. It can be seen that they change symbatically, which is characteristic of surface erosion. Over 168 h, the APET sample lost almost 90% of its mass. It was noted that after 96 h, the pH of the reaction medium decreased to 4.6, which is associated with the release of phthalic acid (phthalates) during the hydrolytic degradation of PET. Such a significant decrease in pH led to the enzyme inactivation and the process rate drop. Returning the pH to its initial value using an alkali solution led to the activity restoration and an increase in the process rate. Further studies were conducted while maintaining the pH of the medium throughout the entire polymer degradation process.

**Figure 5:**
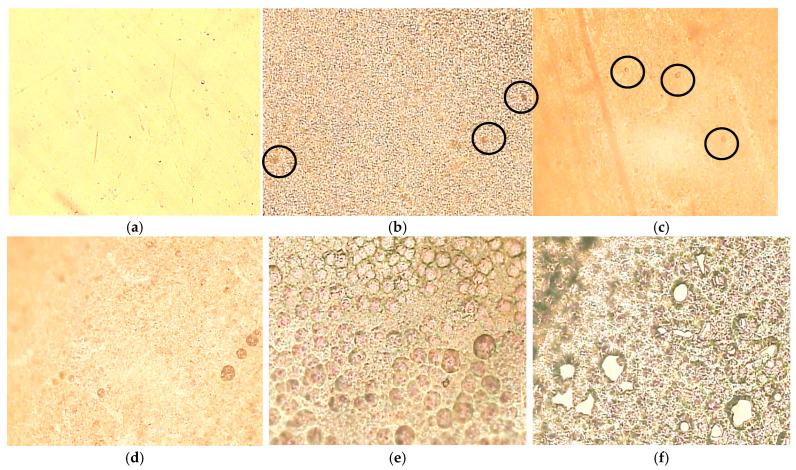
Optical micrographs of the APET film after treatment with LCC^ICCG^ PETase EP 0 (**a**), 24 (**b**), 48 (**c**), 96 (**d**), 144 (**e**), and 168 (**f**) hours at 60 °C. The areas where cavities are detected are circled. The magnification of the images is 100×.

Figure 5 presents images of the APET film after different durations of treatment with LCC^ICCG^ PETase EP solution. It is evident that within just 24 h, cavities with diameters of up to 10–15 µm appear on the surface. Additionally, due to crystallization processes in the near-surface layers, the image becomes grainy as a result of scattering on crystallites. With increasing treatment time, the size of the cavities on the film surface grows to 50–60 µm, and the cavities deepen while their concentration increases. Through-holes are already observed in the sample after 168 h.

The formation of cavity-like defects during the degradation process on the surface of the PET film affects its mechanical properties. As the processing time increases, the elongation at break of the polymer samples decreases, and the statistical scatter of these values increases. After 96 h, the elongation at break ranges between 15–40%. Meanwhile, the strength characteristics remain at the level of 40–45 MPa, and only after 144 h of processing does the mechanical stress at break decrease by more than twofold, reaching 17 MPa.

#### 3.2.3. The Effect of pH on Enzymatic Degradation and Properties of APET

**Figure 6:**
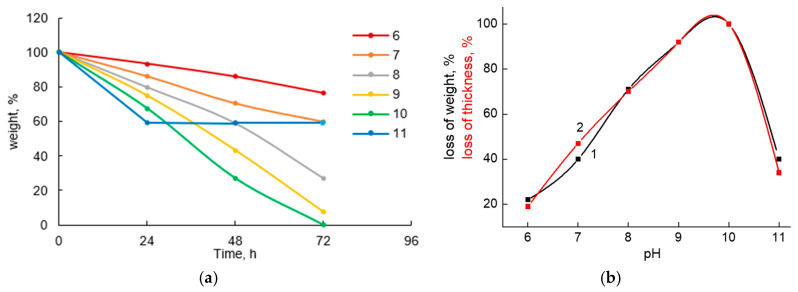
(**a**) Dependence of weight of PET samples on the incubation time in different pH solutions in the presence of LCC^ICCG^ PETase EP at 60 °C. (**b**) Dependence of loss of weight (1) and thickness (2) of APET film on pH at 60 °C in the presence of LCC^ICCG^ PETase EP after 72 h of incubation.

It is known that the catalytic activity of an enzyme is typically maximal within a specific, relatively narrow pH range. Initial experiments showed that the rate of hydrolytic degradation of PET in the presence of LCC^ICCG^ PETase significantly decreases when the pH of the drops from 8 to 4.6. The effect of pH on the hydrolysis rate of APET was more closely studied between 5 and 11 in the presence of LCC^ICCG^ PETase (60 °C, 72 h). Figure 6a presents the dependence of the weight of the samples on time at different pH values. It can be seen (Figure 6b) that at pH 6, the film lost only 20% over 72 h. As the pH increases, the process rate also increases. The maximum weight loss rate was observed at pH 10, under which conditions the sample completely dissolved within 72 h. However, at pH 11, the process rate sharply decreased, which may be associated with enzyme deactivation. The weight loss of APET under such conditions occurs mainly due to hydrolytic degradation catalyzed by the base. It should be noted that control experiments performed at pH 6–11 in the absence of enzyme showed no significant PET weight loss during the 24 h incubation period. Therefore, non-enzymatic alkaline hydrolysis was negligible under the conditions used in this study. The background contribution observed in the corresponding controls was taken into account in the analysis of PET degradation, indicating that the measured effect predominantly resulted from enzymatic hydrolysis.

A similar trend is observed for the reduction in the thickness of the polymer film, indicating that degradation proceeds via a surface erosion mechanism at any pH value in the range of 6 to 11.

**Figure 7:**
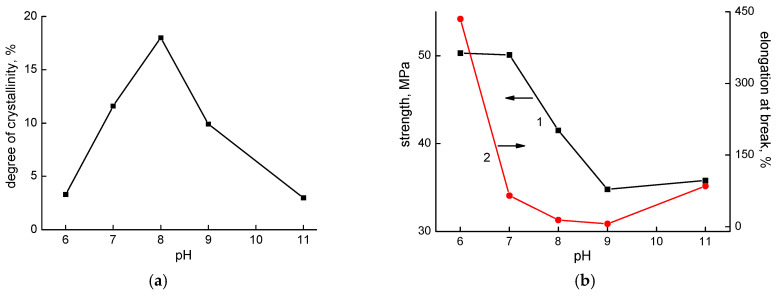
Dependencies of (**a**) the degree of crystallinity and (**b**) the strength (1) and elongation at break (2) of the APET film on the pH in solutions LCC^ICCG^ PETase EP (60 °C, 72 h).

DSC analysis of thermophysical properties of APET samples demonstrated that after incubation with PETase EP (60 °C, 72 h) as the pH of the solution increases, the low-temperature crystallization temperature gradually decreases from 140 to 130 °C. At the same time, the enthalpy of crystallization remains practically independent of the reaction conditions, averaging 26.2 ± 1.6 J g^−1^. However, the enthalpy of melting and the degree of crystallinity exhibit a maximum at pH 8 (Figure 7a). The highest degree of crystallinity reaches 18%. It can be assumed that a more alkaline environment (pH ≥ 9) inhibits PET crystallization due to the potential hydrolysis of ester bonds and the formation of charged carboxyl groups -COO^−^. Previous studies [38,39] have reported that treating even highly crystalline PET samples with an aqueous alkaline solution leads to a decrease in their degree of crystallinity.

Changes in the mechanical properties of PET after treatment with LCC^ICCG^ PETase EP also depend on the pH of the solutions. In a weakly acidic solution (pH 6), the strength characteristics of the film remained almost unchanged after 72 h (Figure 7b)—the strength was 50 MPa, and the elongation at break exceeded 400%. In a neutral solution (pH 7), the elongation at break significantly decreased to 65%, and after the reaction in an alkaline solution (pH 8–9), the polymer samples became sharply brittle. Their strength decreased to 30–40 MPa, and elongation dropped to 10–15%. Thus, the activity of the enzymatic preparation is highest at pH 8–10. In acidic solutions with pH ≤ 6 and strongly alkaline solutions with pH ≥ 11, the activity of PETase is low, and the degradation process of PET proceeds at slower rates.

#### 3.2.4. HPLC Analysis of PET Hydrolysis Products

To determine the composition of soluble hydrolysis products, PET samples were subjected to enzymatic hydrolysis and the reaction mixtures were analyzed by HPLC. Aliquots were collected after 1, 2, and 3 days of incubation to monitor product formation. Terephthalic acid (TPA), mono(2-hydroxyethyl) terephthalate (MHET), and bis(2-hydroxyethyl) terephthalate (BHET) were used as external standards for peak identification and quantification. Terephthalic acid was identified as the predominant hydrolysis product. HPLC chromatograms (Figure S2; UV detection at 237 nm) showed a major peak corresponding to the TPA standard. Quantitative analysis demonstrated that TPA accounted for 88–95% of the detected hydrolysis products (excluding free ethylene glycol), depending on the enzyme loading and hydrolysis time, whereas MHET was present only in minor amounts. BHET was detected only transiently during the early stages of hydrolysis and disappeared upon further incubation. The low accumulation of intermediate products indicates that the enzyme preparation efficiently catalyzes PET depolymerization to its final monomeric products.

The minor accumulation of these intermediates indicates that the catalytic system effectively facilitates complete depolymerization to monomer.

### 3.3. Effect of PET Crystallinity on Its Degradation in the Presence of LCC^ICCG^ PETase

In the preliminary stage of the study, film samples of PET with varying degrees of crystallinity were prepared. Figure S3 shows the dependence of the polymer’s degree of crystallinity on the annealing time at a temperature of 110 °C. In this case, an S-shaped kinetic curve was obtained, which is characteristic of a crystallization process with homogeneous nucleation. For this system, the induction period was approximately 15 min, and after 45–60 min, the crystallization process was practically complete. The formation of the crystalline phase leads to the clouding of the samples, and samples with a crystallinity degree of about 40% become white in color.

All the initial samples with varying degrees of crystallinity underwent deformation with neck formation and were characterized by high elongation at break values of 400–680%. At the same time, the increase in crystallization time led to a slight rise in the elastic modulus to 1.6 GPa and the yield strength to 70 MPa, as well as a reduction in elongation at break compared to the amorphous sample.

**Figure 8:**
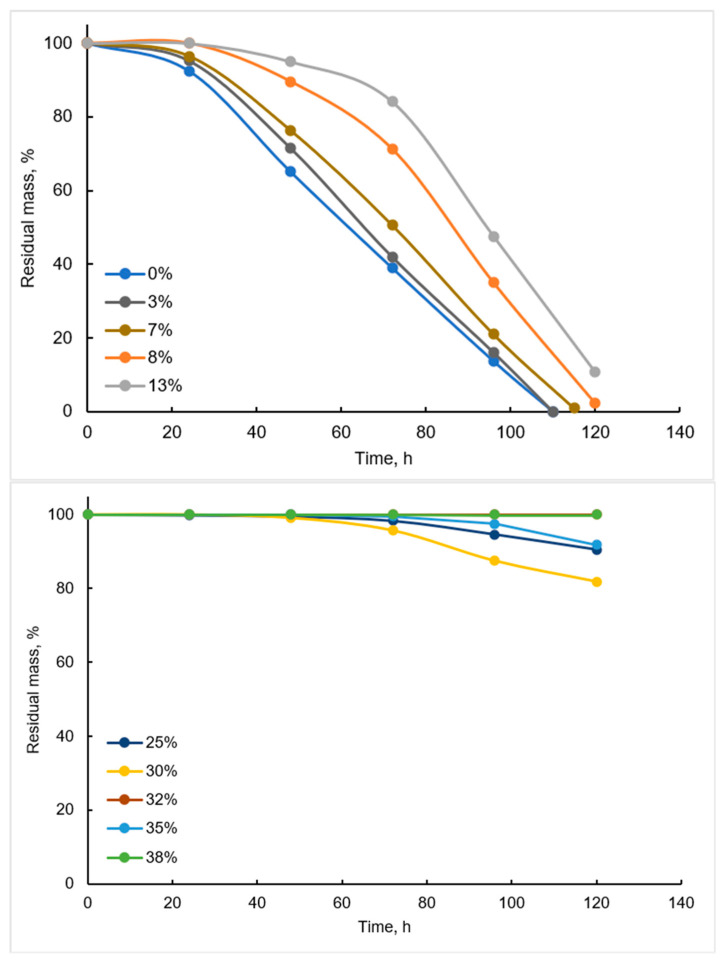
Dependence of the weight of PET samples with varying degrees of crystallinity on the incubation time in the presence of LCC^ICCG^ PETase (60 °C, pH 8).

The study examined the kinetics of weight loss in PET samples with varying degrees of crystallinity (Figure 8) during treatment with LCC^ICCG^ PETase EP at 60 °C and pH 8. It is evident that as the degree of crystallinity increases, the hydrolysis rate decreases. Even the formation of a small fraction of the crystalline phase (less than 10%) leads to a noticeable reduction in the weight loss rate, accompanied by an induction period of approximately 24 h. Samples with a crystallinity degree of less than 15% completely lose their mass within about 110–130 h. For samples with a crystallinity degree above 30%, the rate of enzymatic degradation significantly decreases, resulting in a weight loss of no more than 20% over 120 h. It was noted that, unlike amorphous samples or those with very low crystallinity (less than 5%), which exhibit a gradual decrease in thickness in parallel with weight loss (Figure 4), semicrystalline samples do not change in thickness but instead transform into a mesh. Additionally, the degree of crystallinity of semicrystalline samples remains practically unchanged during degradation.

**Figure 9:**
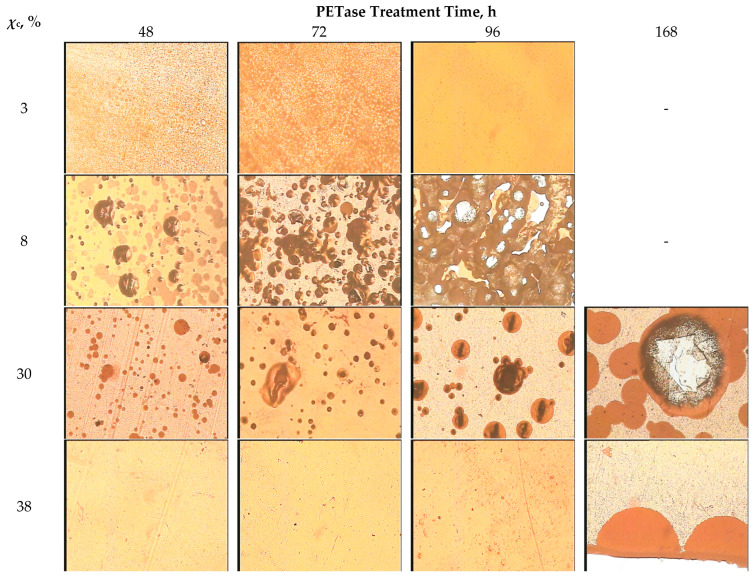
Optical micrographs (50× magnification) of PET films with varying degrees of crystallinity and different incubation times in PETase EP at 60 °C and pH 8.

A study using optical microscopy to examine the evolution of the morphology of PET films with varying degrees of crystallinity during treatment with LCC^ICCG^ PETase revealed (Figure 9) that the degradation of samples with a very low degree of crystallinity (less than 5%) occurs similarly to that of amorphous samples.

**Figure 10:**
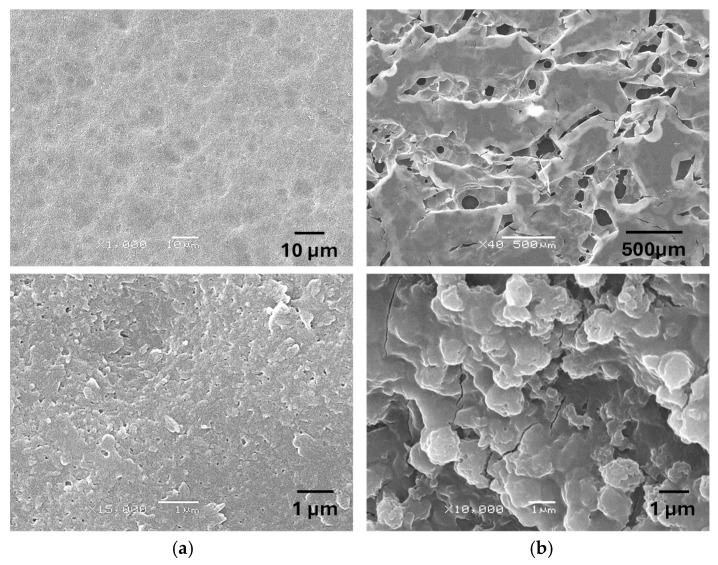
SEM micrographs at two magnifications of the surface of PET films with crystallinity degrees of 3% (**a**) and 8% (**b**) after incubation with LCC^ICCG^ PETase EP (60 °C, pH 8, 96 h).

In this case, the degradation process occurs simultaneously across the entire surface area, accompanied by the formation of numerous shallow caverns several microns in size, containing submicron-sized pores (Figure 10a), and a gradual reduction in the sample thickness. During the degradation of semicrystalline samples with a higher degree of crystallinity, large individual cavities, primarily circular and hundreds of microns in size, form, likely at defect sites. These cavities deepen over time (Figure 10b) and eventually merge. It can be assumed that in this case, weight loss occurs due to the degradation of localized regions penetrating into the depth of the film. For samples with the highest degree of crystallinity, the degradation process begins at edge defects, forming semicircular degradation zones (Figure 9).

**Figure 11:**
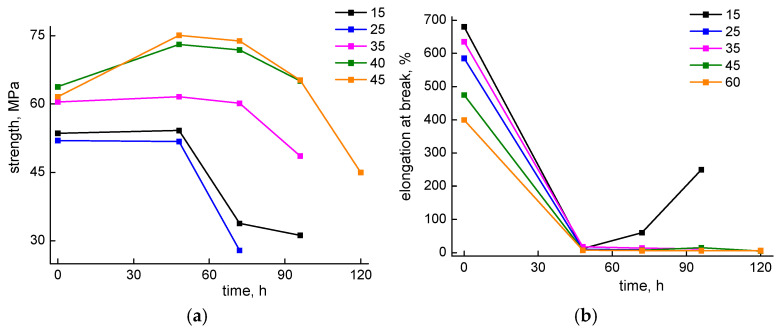
Dependencies of (**a**) strength and (**b**) elongation at break of PET films with different annealing times at 110 °C (15 min—3.5%, 20 min—8%, 25 min—13%, 35 min—25%, 40 min—32%, 45 min—35%, 60 min—38%) on the incubation time in LCC^ICCG^ PETase EP solution (60 °C, pH 8).

The formation of defects during degradation leads to a decrease in the mechanical parameters of the polymer material. Figure 11 presents the dependencies of the strength characteristics of PET films with different annealing times (varying in crystallinity), on the treatment time with LCC^ICCG^ PETase. It is evident that the strength of samples with a low degree of crystallinity (less than 15%) begins to decrease after 72 h of treatment, while for samples with a crystallinity degree above 25%, the decrease occurs after 96 h of enzyme treatment. The elongation at break sharply decreases from 400–700% to 10–20% after just 48 h of incubation due to the appearance of surface defects. Notably, for samples with a crystallinity degree of <5%, the elongation at break increases again at longer degradation times, which may be associated with an increase in the concentration of small surface defects and their smoothing. This is in sharp contrast to the degradation of PET samples with a higher degree of crystallinity, for which the depth of defects only increases over time, eventually leading to the formation of through-holes. As a result, the elongation at break decreases to 5% after 96–120 h of the process.

Thus, the study demonstrated that the presence of the crystalline phase leads to a reduction of the enzymatic hydrolysis rate of the ester bonds catalyzed by LCC^ICCG^ PETase. Additionally, the nature of the process itself changes. Relatively large cavities appear on the surface of the semicrystalline films which deepen over time. This results in the overall thickness of the sample remaining unchanged, while the sample itself acquires a porous, mesh-like structure. The only exception is samples with a very low degree of crystallinity (<5%), whose behavior is similar to that of amorphous films, meaning their degradation follows a surface erosion mechanism with a gradual reduction in thickness, without affecting the bulk. The disparity in the behavior of PET samples with different degrees of crystallinity can be attributed to the fact that the ester bonds in the crystalline regions of the polymer are inaccessible to the enzyme due to steric hindrance, conformational constraints, and low chain mobility. Therefore, in semicrystalline samples, only the amorphous regions and imperfect crystallites are susceptible to enzymatic degradation. This results in a localized degradation process, where the enzyme gradually breaks down regions of the accessible polymer material, creating localized channels (cracks and pores). The crystalline phase, however, remains relatively intact and forms nodes of a porous three-dimensional network. This degradation behavior fundamentally differs from that observed in amorphous or low-crystallinity films, which undergo surface erosion accompanied by a gradual decrease in thickness. At the same time crystalline regions appear in amorphous films during the enzymatic hydrolysis process. However, these regions are limited to the surface layers and do not prevent the complete degradation of the samples in the presence of LCC^ICCG^ PETase EP.

## 4. Conclusions

This study investigates changes in the mechanical properties and structure of PET during enzymatic degradation induced by a novel recombinant enzyme preparation containing LCC^ICCG^ PETase, obtained through expression of a chimeric genetic construct in an ascomycete *P. verruculosum*. It was shown that the employed approach enables high expression of the target protein.

The enzymatic effect of LCC^ICCG^ PETase on the changes in mechanical properties and structure of PET was studied using films with varying degrees of crystallinity. It was shown that after treatment with the LCC^ICCG^ PETase enzyme preparation, a decrease in the mechanical properties of PET was observed: the yield stress and plateau decreased by 20–25%, tensile strength decreased approximately twofold, and elongation at break decreased by 5–10 times. In the presence of the enzyme preparation, the deformation mechanism involves neck formation; however, earlier failure is associated with surface defects due to enzymatic degradation rather than bulk material disintegration.

Microscopy data corroborated the surface-localized effect of LCC^ICCG^ PETase, revealing the formation of cavities and fissures containing submicron-scale pores. Analysis of the process kinetics demonstrated a concerted reduction in both sample mass and thickness, which, after 168 h of incubation, constituted 75% and 90% of their initial values, respectively.

Enzymatic degradation under acidic pH proved ineffective. Increasing the pH from 6 to 10 significantly accelerated the weight loss rate without loss of enzyme activity. The application of alkaline conditions (pH ≥ 9) is further advantageous as it mitigates the increase in crystallinity of APET samples, diminishes their strength parameters, and ultimately induces embrittlement.

Investigation of the effect of crystallinity degree showed that while complete destruction of samples with *χ*_c_ < 13% is possible within a reasonable time, at *χ*_c_ > 30% weight loss over the same period is either negligible or absent. DSC confirmed an increase in crystallinity degree in the surface layer for weakly crystalline samples due to crystallite formation, while for highly crystalline samples, optical and SEM microscopy revealed the formation of a mesh structure or edge defects. Mechanical testing demonstrated that a lower degree of crystallinity correlates with a more rapid decline in strength— for values below 15%, after 72 h, and for values above 25%, after 96 h of enzymatic treatment. Within just two days of incubation, due to surface defects, the elasticity of the material sharply drops from 400–700% to 10–20%.

## Figures and Tables

**Table 1:** Characteristics of LCC^ICCG^ PETase EPs: enzymatic activity, total protein concentration, and LCC^ICCG^ PETase content. Values are presented as mean ± SD (*n* = 3).

Linker	EP	Enzyme Activity (pNPB), U/g	Protein, mg/g	PETase, %
**Flexible**	FLPET1	63,900 ± 200	440 ± 10	20.5
	FLPET2	98,700 ± 500	460 ± 20	19.7
**Rigid**	RLPET1	2550 ± 90	170 ± 10	1.6
	RLPET2	21,200 ± 100	250 ± 10	6.3

**Table 2:** Results of DSC of APET film treated with different enzyme preparations.

Enzyme Preparation, mg of Total Protein mg^−1^ PET	*T*_g_, °C	*T*_cc_, °C	∆*H*_cc_, J g^−1^	*T*_m_, °C	∆*H*_m_, J g ^−1^	χ_c_, %
w/o treatment (initial film)	73	144	32.8	247	34.2	1
	30 °C, pH 8, 24 h					
0.1 M Tris-HCl	75	147	33.3	248	33.5	0
B1, 0.75	75	148	35.8	248	34.0	0
B1, 2.25	75	149	33.7	248	38.1	3.1
PETase, 0.75	74	147	33.9	247	39.7	4.1
PETase, 2.25	74	146	30.5	248	38.1	5.4
	60 °C, pH 8, 24 h					
0.1 M Tris-HCl	75	137	31.5	248	33.7	1.6
B1, 0.75	75	139	32.2	247	30.0	0
B1, 2.25	76	136	31.2	248	36.8	4.0
PETase, 0.75	75	136	29.5	247	43.4	9.9
PETase, 2.25	73	137	29.9	248	42	8.6
	60 °C, pH 8, 72 h					
PETase, 0.92	79	137	30.5	248	49.5	13.5
PETase, 1.55	80	138	29.6	248	37.3	5.5

**Table 3:** Mechanical testing data.

Enzyme Preparation, mg of Total Protein mg^−1^ PET	Elastic Modulus, MPa	Yield, MPa	Plateau, MPa	Stress at Break, MPa	Elongation at Break, %
w/o treatment (initial film)	1190 ± 120	51.4 ± 1.6	40.4 ± 0.2	61.5 ± 1.1	690 ± 20
	30 °C, pH8, 24 h				
Air	1360 ± 40	54.7 ± 2.6	40.3 ± 0.2	58.8 ± 5.0	660 ± 80
0.1 M Tris-HCl	1500 ± 90	50.7 ± 1.1	39.7 ± 0.4	61.9 ± 1.9	680 ± 20
B1, 0.75	1410 ± 50	50.5 ± 0.9	38.7 ± 0.4	56.5 ± 2.4	635 ± 25
B1, 2.25	1480 ± 90	52.4 ± 2.1	39.6 ± 0.3	59.3 ± 2.7	650 ± 50
PETase, 0.75	1550 ± 40	53.6 ± 2.1	40.8 ± 0.2	56.9 ± 3.6	600 ± 60
PETase, 2.25	1510 ± 40	56.3 ± 2.0	40.9 ± 0.4	57.1 ± 5.4	600 ± 70
	60 °C, pH8, 24 h				
Air	1220 ± 40	56.4 ± 2.3	41.3 ± 0.5	57.1 ± 5.6	600 ± 90
0.1 M Tris-HCl	1310 ± 60	44.9 ± 3.4	40.0 ± 0.4	61.8 ± 3.8	660 ± 50
B1, 0.75	1030 ± 100	44.7 ± 2.7	40.6 ± 1.4	61.1 ± 3.9	690 ± 50
B1, 2.25	1120 ± 40	43.1 ± 0.6	39.7 ± 1.7	59.5 ± 3.1	660 ± 40
PETase, 0.75	950 ± 110	32.8 ± 0.6	30.2 ± 0.6	29.7 ± 0.6	50 ± 20
PETase, 2.25	1010 ± 80	36.2 ± 1.5	30.4 ± 0.3	30.5 ± 0.2	130 ± 60
	60 °C, pH8, 72 h				
PETase, 0.92	1250 ± 50	50.8 ± 2.2	42.2 ± 3.3	42.2 ± 3.4	70 ± 20
PETase, 1.55	1420 ± 110	52.8 ± 1.0	44.4 ± 1.2	44.4 ± 4.2	90 ± 15

## Data Availability

Data available in Supplementary Materials.

## Supplementary Materials

The following supporting information can be downloaded at: https://www.mdpi.com/article/10.3390/polym18121510/s1, Figure S1: SDS-PAGE analysis of the purified recombinant PETase (Lane M—molecular weight marker (kDa)); Figure S2: HPLC chromatogram of APET hydrolysis products; Figure S3: Dependence of the degree of crystallinity of the PET film on the annealing time in air at 110 °C.

## Abbreviations

The following abbreviations are used in this manuscript:

BHET	bis(2-hydroxyethyl) terephthalate
CBHI	Cellobiohydrolase I
DSC	Differential scanning calorimetry
EP	Enzyme preparation
HPLC	High-performance liquid chromatography
LCC	Leaf-branch compost cutinase
MCC	Microcrystalline cellulose
MHET	Mono(2-hydroxyethyl) terephthalate
PET	Polyethylene terephthalate
pNPB	p-Nitrophenyl butyrate
TGA	Thermogravimetric analysis
TPA	Terephthalic acid
