# Supplementary figures and images for: Changes in Mechanical Properties and Structure of PET Films Treated with Metagenome-Derived LCCICCG PETase Heterologously Expressed in Penicillium verruculosum
Source: Polymers (Basel). 2026 Jun 17;18(12):1510. doi: 10.3390/polym18121510 (PMC13306724; doi:10.3390/polym18121510)

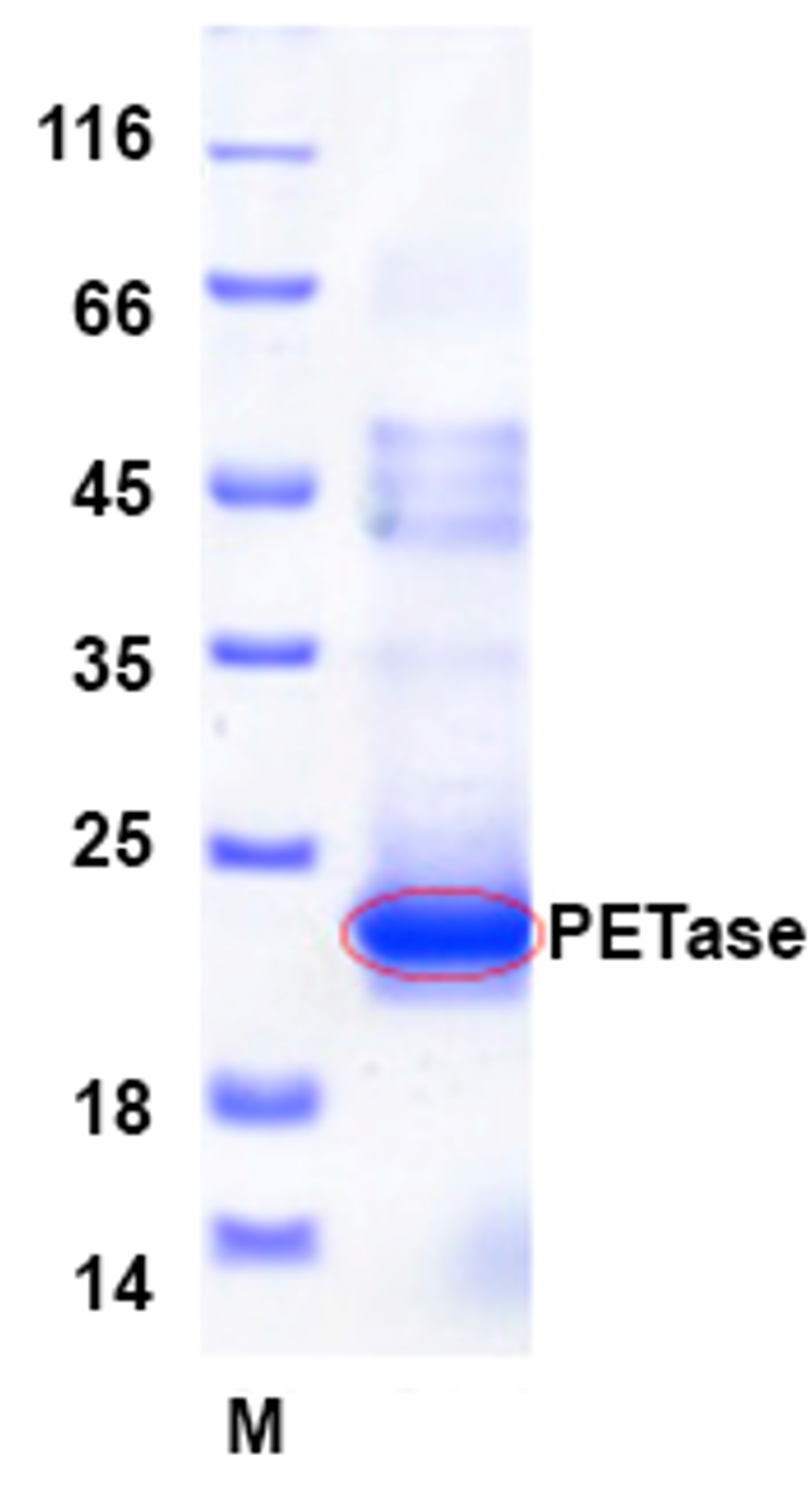

Supplement: Supplementary file 1 [file polymers-18-01510-s001.zip › Figure S1.png]

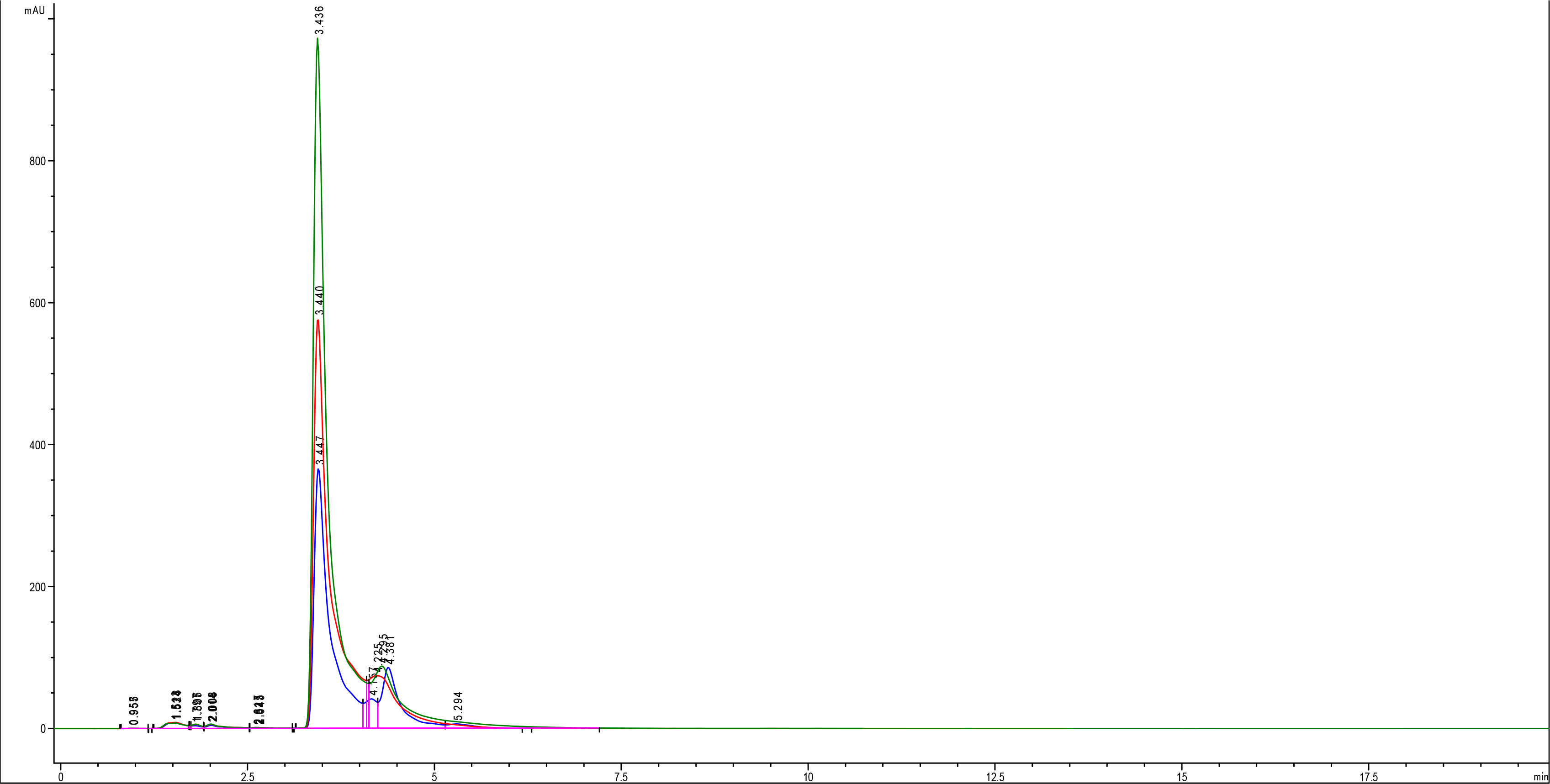

Supplement: Supplementary file 1 [file polymers-18-01510-s001.zip › Figure S2.png]

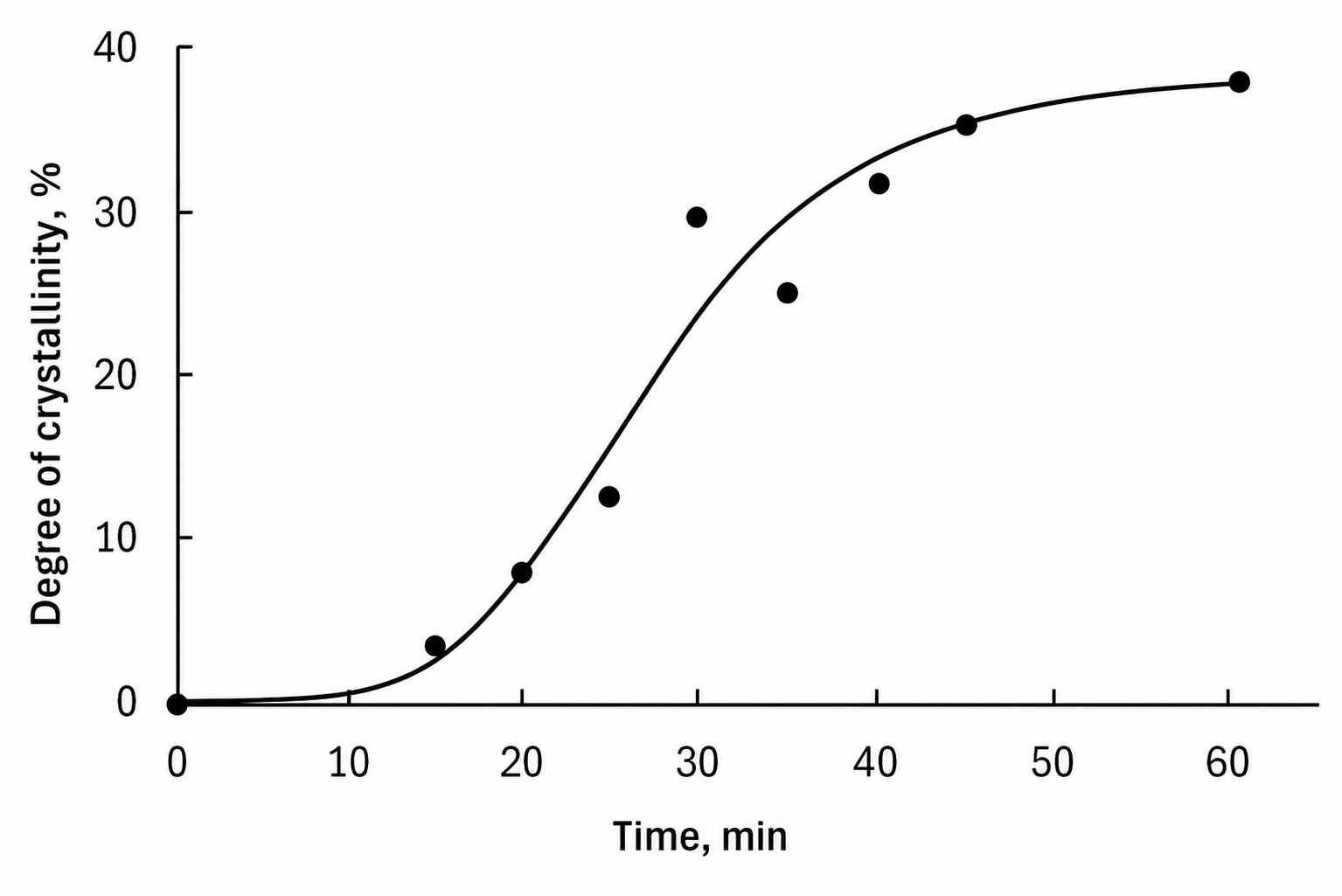

Supplement: Supplementary file 1 [file polymers-18-01510-s001.zip › Figure S3.png]
